# Does Your Organization Assess Risk Culture? If Not, It Should. Here's How

**DOI:** 10.3389/frma.2022.891324

**Published:** 2022-05-23

**Authors:** Paul Miller

**Affiliations:** National Institutes of Health, Office of Management Assessment, Bethesda, MD, United States

**Keywords:** risk, risk culture, ERM, risk management, behavioral metrics

## Abstract

Risk culture is an important subset of organizational culture that Enterprise Risk Management (ERM) program managers should assess. Insights into the organization's risk culture can be found in behavioral metrics. Valuable behavioral metrics are present in workforce climate assessments such the Federal Employee Viewpoint Survey (FEVS). Training evaluation frameworks, such as the Kirkpatrick Model, set the foundation for collecting and analyzing behavioral metrics. These sources of behavioral metrics should be utilized in making ERM program decisions, especially regarding risk management policies, training, and communications. A good risk culture assessment offers clues into the risk mentality of decision makers in the organization. ERM program managers would serve their organizations well by knowing the risk mentality of people before important decisions are made.

## Introduction

“Culture eats strategy for breakfast” (Strategies for Influence, 2021). This phrase has been attributed to a famous management consultant named Peter Drucker and is popular at various leadership, change management, or other business conferences and forums. It conveys a simple message. The best organizational strategies can be rendered ineffective by the organization's culture. Enterprise Risk Management (ERM) practitioners know just how important organizational culture is to the success of ERM programs. This is why behavioral metrics should be utilized to assess the organizational risk culture.

## What Is “Risk Culture?”

According to the Committee of Sponsoring Organizations of the Treadway Commission (COSO), “risk culture pertains to ethical values, desired behaviors, and understanding of risk in the entity” (Committee of Sponsoring Organizations of the Treadway Commission, 2017). How much risk are people in an organization allowed to take in the performance of their duties? Is the organization, or subgroups within, risk seeking or risk adverse? When, how, and to whom should risks be communicated? Are people punished for communicating risks? Are people who take risks smartly, but still end up failing, rewarded or punished? The answers to these questions constitute the fabric of an organization's risk culture. The responses and behavior of senior leaders, middle management, and frontline personnel concerning the questions above are far more indicative of the risk culture than any number of policies and memorandums. ERM program managers should assess their organization's risk culture periodically because risk culture influences risk management effectiveness. Risk culture affects decisions such as setting strategic objectives, risk appetites (how much risk to take to achieve objectives), and willingness to conform to compliance requirements (Hillson, 2013). Assessing risk culture will provide insights into the mindset of decision makers BEFORE these important decisions are made.

## Assessing Risk Culture With FEVS Behavioral Metrics

The Federal government annually administers the Federal Employee Viewpoint Survey (FEVS) to gauge the federal workforce climate (Office of Personnel Management, 2021). The FEVS survey focuses on employee engagement, organizational leadership, and work experiences. Months after the survey concludes, agency leadership is provided a summary of how their employees (anonymously) answered each question plus a comparison to the responses provided by employees of similar agencies. Based on these responses and comparative data, agency leaders may change how they manage human capital programs such as leave (paid time off), promotions, workplace flexibilities, and training opportunities. While the FEVS provides useful behavioral metrics for an organization's culture, it also sheds light into the risk culture of the organization as well. Not every FEVS question pertains to risk culture, but there are some that uncover the risk mindset of employees. The FEVS questions below are indicators of the organizational risk culture. Participants answer how strongly they agree or disagree with these statements.

- I can disclose a suspected violation of any law, rule, or regulation without fear of reprisal.
- Employees are protected from health and safety hazards on the job.
- My agency is successful at accomplishing its mission.
- Managers communicate the goals of the organization.

**Figure 1:**
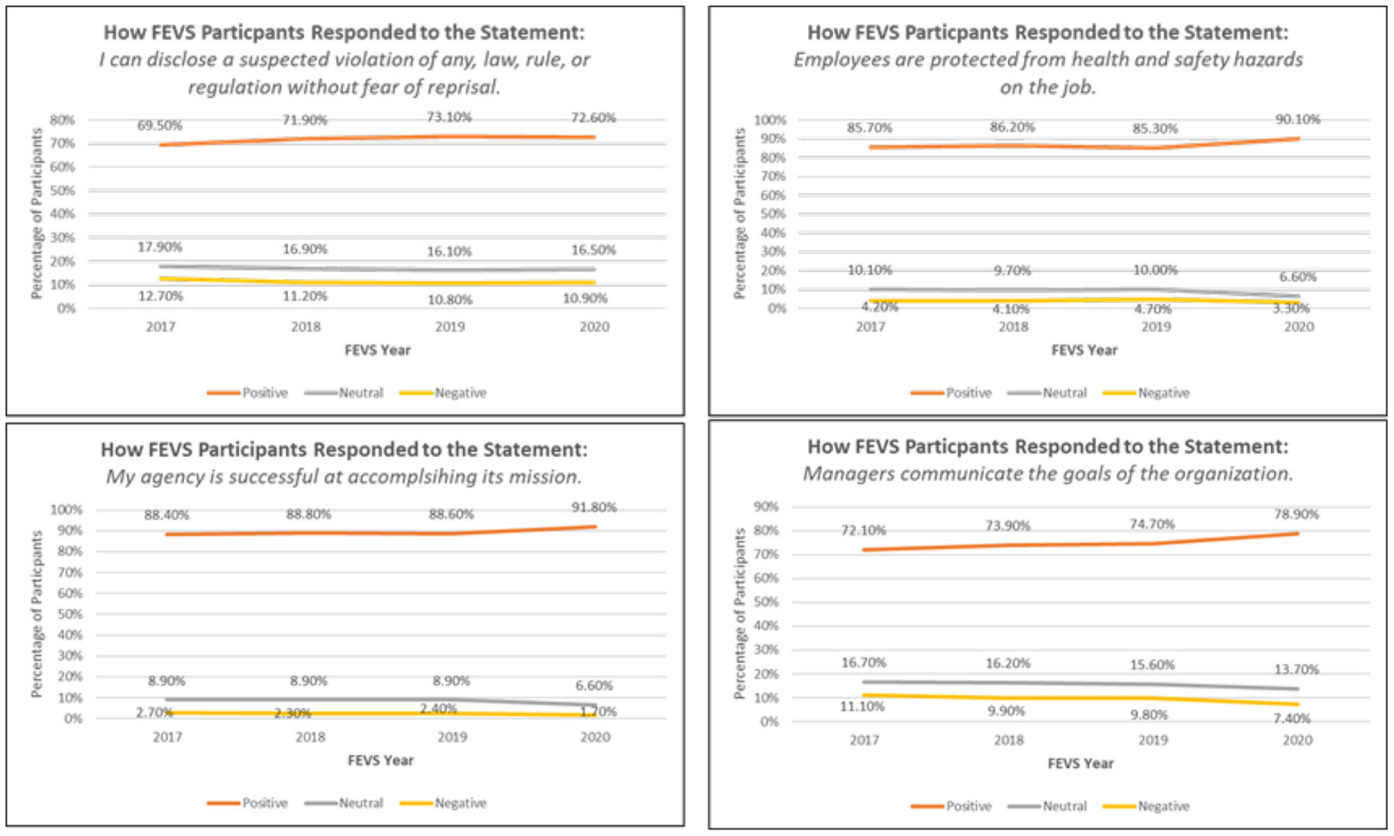
FEVS results for 2017–2020 for risk culture questions (Data visuals created by author).

Over the past 4 years, the National Institutes of Health (NIH) Risk Management Program has been tracking the percentages of respondents who answer positively (Strongly Agree/Agree), neutral, and negatively (Strongly Disagree/Disagree) to these statements. The 2017–2020 FEVS results have established a baseline for each of these measures so that the NIH Risk Management Program can monitor these FEVS responses for undesirable trends or significant deviations from those baselines. Figure 1 below shows the percentage of responses that were positive, neutral, and negative for the FEVS risk culture questions identified above between calendar years 2017–2020. In 2020, nearly 73% of NIH FEVS participants responded positively to the question regarding the disclosure of a suspected violation of law without reprisal. Over the past couple of years, the positive response rate to this question has hovered around the 72–73% mark. The goal of the NIH Risk Management Program is to get as close to 100% as possible because risks cannot be identified if people feel like they cannot disclose violations without reprisal. If the percentage of participants that answer positively to this question begins to drop (or the percentage of respondents that answer negatively begin to rise) then this indicates that the NIH risk culture may be prohibiting risk identification.

If there were to be undesired changes in the above measures, the NIH Risk Management Program may tailor risk identification methods. For example, the NIH Risk Management staff could collect risk data by conducting anonymous surveys or nonattributable interviews. Additionally, the NIH Risk Management Program could host training sessions or webinars for supervisors and managers about the importance of allowing employees to identify risks without fear of reprisals. Another option could be to draft an e-mail or memorandum from the NIH Director reminding supervisors and managers that frontline staff are essential to proactively identifying and managing risks. Without measuring and monitoring FEVS questions like the ones above, the NIH Risk Management Program would not know that such activities were needed. These FEVS measures inform how the NIH Risk Management Program should respond to changes in NIH employee behavior so that NIH's Risk Management Program objectives can be achieved.

## Utilizing Behavior-Based Training Metrics to Assess Risk Culture

In addition to the FEVS questions listed above, NIH assesses risk culture using behavioral metrics derived from NIH's risk management training activities. The goal of risk management training is to provide NIH employees with the knowledge, skills, and attitudes necessary to apply risk management principles to their jobs. How is training, especially risk management training, measured to ensure that successful training outcomes such as certain desired risk management behaviors are increasing? The Kirkpatrick Model provides a framework for measuring training programs (Kirkpatrick, 1994). The NIH Risk Management Program uses the Kirkpatrick Model because it focuses on employee behaviors. The Kirkpatrick Model aligns employee behaviors acquired or reinforced through training activities to organizational goals. The Kirkpatrick Model identifies four levels of training evaluations (Kirkpatrick, 1994).

- Level 1 (Reaction): Evaluate how employees felt about the training.
- Level 2 (Learning): Evaluate to what degree did the employees retain or absorb training.
- Level 3 (Behavior): Evaluate to what degree are the employees applying the training.
- Level 4 (Results): Evaluate influence on organizational objectives.

These four levels provide the framework for collecting and utilizing behavioral metrics to inform NIH's Risk Management Program operations. These behavioral metrics are gleaned from post-training surveys, stakeholder surveys, and analysis of job-related products.

The first level of the Kirkpatrick Model deals with the employees' emotional response to the training. It's not surprising that a person's emotions and behaviors are connected, so gathering this type of response is crucial. The NIH Risk Management post-training survey asks employees behavioral questions like, “Would you recommend this training to a colleague?” This type of question is an important behavioral indicator because NIH's Risk Management Training is not mandatory. When NIH markets risk management training, it relies on employees spreading their positive experiences with their colleagues.

Level 2 behavioral metrics focuses on whether the employees absorbed the training material. Typically, this is done through quizzes, progress checks, and test; however, these types of measuring instruments do not really get to the behavior of employees. Instead of tests, employees are asked after a training session to identify what knowledge or skills from the training session they intend to apply to their job. This is critically important in risk management because knowing risk management is simply not enough to identify and manage organizational risks. Employees must be able to apply risk management principles and concepts to their daily work. Risk management application varies from someone in the human capital, finance, grants management, or scientific research arenas. If employees cannot apply the risk management training, then the NIH Risk Management Program is wasting time and resources.

Level 3 is directly tied to employee behavior. In most organizational settings, on-the-job performance is a behavioral indicator of training effectiveness. For the NIH Risk Management Program, analysis of annual risk inventories^1^ provides insights into the behaviors of NIH's risk management practitioners. The primary focus of the NIH risk management training curriculum is to get risk management practitioners to complete annual risk inventories according to prescribed data quality standards. For NIH, risk inventories are a list of each institute or center's risks and risk responses. The prescribed data quality standards for these risk inventories ensures that the NIH Risk Management Program receives useful risk data. For example, the NIH Risk Management Program requires risks to be written in an “If/Then” format (If X happens, then Y will occur.) so that the risk is clearly and concisely communicated. Additionally, reduce risk responses must identify future mitigation activities and have a target date for those actions. Every year, the NIH Risk Management Program analyzes the risk inventories from the 27 institutes and centers to determine how well they are adhering to the data quality standards. This analysis informs future risk management training activities.

Finally, level 4 evaluates how risk management training influences risk management at NIH. The FEVS risk culture behavioral questions are also helpful here–especially the question about managers communicating the goals of the organization. It's impossible to identify relevant risks if risk practitioners do not know the goals (and the associated objectives) of the organization. Part of the NIH Risk Management Program training describes how to integrate strategic planning and risk management activities. This FEVS question helps inform the efficacy of that training. Additionally, the NIH Risk Management Program conducts stakeholder surveys to determine if decision-makers regularly incorporate risk management information into management decisions such as budget and program performance. If decision-makers tell the NIH Risk Management Program that risk information is not incorporated into the process, then changes need to be made on what and/or how risk data is being communicated to decision makers.

Metrics collected utilizing the four levels of the Kirkpatrick Model provide behavioral insights into NIH's risk culture. For example, analysis of past risk inventories [Level 3 (Behavior)] showed a downward trend of risk statements that were in an “If/Then” format and written clearly and concisely. Furthermore, the analysis of risk inventories revealed that the majority of NIH's risk responses were identifying past mitigation actions instead of identifying avenues for future actions. This demonstrates a lack of risk understanding, which as noted earlier is essential to risk culture. According to a NIH internal 2020 risk management stakeholder survey [Level 4 (Results)], roughly 27% of participants do not believe that (or do not know if) risk management information is being used in organizational decision making. This demonstrates that some organizations within NIH are not applying the risk management principles being taught. This indicates an ineffective risk culture in some areas within NIH.

These training metrics served as the catalyst for several changes in the NIH Risk Management Program to improve NIH's risk culture. First, the risk management training shifted focus from online learning modules to classroom and webinar case-based sessions. Two of the risk management training courses require employees to assume the role of a risk management practitioner at a fictitious organization where they are presented with real-world scenarios. This provided a risk-free environment to apply risk management principles to similar job situations. Second, the Risk Management Program constructed a course and training video on how to write proper “IF/Then” risk statements. The training breaks down the requirements of the “IF/Then” risk statement, offers examples, and provides an opportunity to practice writing risk statements with real-world risk data. Finally, the NIH Risk Management Program restructured its “Functional Owner Report” to deliver more useful risk data. Functional Owner Reports are annual risk data reports that the NIH Risk Management Program offers to business and program area leaders in NIH. These newly revamped reports contain additional risk trend data that could assist these key NIH decision makers. Hopefully, these changes will improve NIH's risk culture indicators in the coming years.

## Risk Culture Assessment Informs ERM Program Decisions

**Figure 2:**
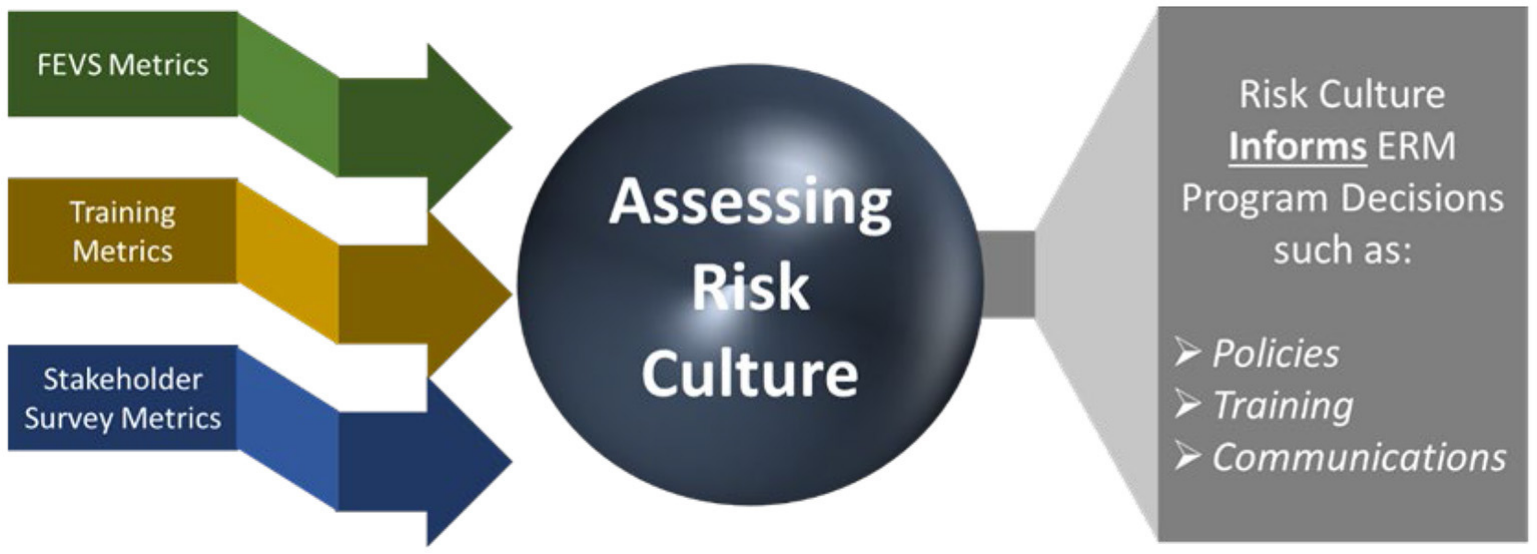
NIH utilizes multiple sources for behavioral metrics to assess risk culture, which informs ERM program decisions. (Graphic created by author).

Figure 2 depicts how the NIH Risk Management Program utilizes behavioral metrics to inform ERM program decisions. The NIH Risk Management Program reviews periodic data from the FEVS, risk management training activities, and other stakeholder surveys to evaluate NIH's risk culture. These metrics provide the behavioral insights to the risk mindset of NIH personnel. Based on the data trends from those metrics, the NIH Risk Management Program decides if changes in ERM policy, training, or communications are warranted. Once the applicable changes are implemented, the next iteration of these metrics are analyzed to determine if the implemented changes had the desired effect. In this manner, the NIH Risk Management Program decisions are more data-driven and aligned with NIH's risk culture.

## Conclusion

Assessing risk culture should be an important aspect of any ERM program. Even the most comprehensive ERM framework requires the support of a positive organizational risk culture. Organizations need to measure the “people side” of risk management. People make decisions that determine the fate of the organization. If an ERM program is only looking at organizational performance metrics to determine effectiveness, then these ERM programs are at risk of only seeing half the picture. Behavioral metrics serve as indicators for ERM program managers to “pulse check” the risk mindset of the people in their organizations. Organizations probably have these behavior metrics readily available in climate assessment, training, and stakeholder surveys. It is just a matter of pulling it all together. Risk culture assessments can lead to more informed ERM program decisions. Do not let a good organizational ERM strategy get gobbled up for breakfast by a ravenous risk culture.

## Data Availability Statement

The data analyzed in this study is subject to the following licenses/restrictions: The article uses Federal Employment Viewpoint Survey data that is archived and may not be available to the public. Requests to access these datasets should be directed to PM, paul.miller3@nih.gov.

## Author Contributions

PM is the sole author of this article and created the accompanying visuals.

## Conflict of Interest

The author declares that the research was conducted in the absence of any commercial or financial relationships that could be construed as a potential conflict of interest.

## Publisher's Note

All claims expressed in this article are solely those of the authors and do not necessarily represent those of their affiliated organizations, or those of the publisher, the editors and the reviewers. Any product that may be evaluated in this article, or claim that may be made by its manufacturer, is not guaranteed or endorsed by the publisher.

## Footnotes

^1^*A “Risk Inventory” is a term the NIH Risk Management Program uses to describe the database of identified risks. “Risk Inventory” is similar to the term “Risk Register” that may be commonly used in other ERM Programs*.
